# ﻿Newly found and rediscovered hornworts (Anthocerotophyta) in Poland: Indicators of climate change impact in Central Europe

**DOI:** 10.3897/phytokeys.248.134729

**Published:** 2024-10-31

**Authors:** Vítězslav Plášek, Lukáš Číhal, Frank Müller, Martina Pöltl, Mariusz Wierzgoń, Ryszard Ochyra

**Affiliations:** 1 Department of Biology and Ecology, University of Ostrava, Chittussiho 10, CZ-710 00 Ostrava, Czech Republic University of Ostrava Ostrava Czech Republic; 2 Institute of Biology, University of Opole, Oleska 48, PL-45-052 Opole, Poland University of Opole Opole Poland; 3 Silesian Museum, Nádražní okruh 31, CZ-746 01 Opava, Czech Republic Silesian Museum Opava Czech Republic; 4 Institut für Botanik, Technische Universität Dresden, D-01062 Dresden, Germany Technische Universität Dresden Dresden Germany; 5 Studienzentrum Naturkunde Joanneum Graz, Weinzöttlstraße 16, AT-8045 Graz, Austria Studienzentrum Naturkunde Joanneum Graz Graz Austria; 6 Institute of Biology, Biotechnology and Environmental Protection, Faculty of Natural Sciences, University of Silesia in Katowice, Jagiellońska 28, PL-40-032 Katowice, Poland University of Silesia in Katowice Katowice Poland; 7 National Collection of Biodiversity, W. Szafer Institute of Botany, Polish Academy of Sciences, Lubicz 46, PL-31-512 Kraków, Poland W. Szafer Institute of Botany, Polish Academy of Sciences Kraków Poland

**Keywords:** *Anthocerosneesii*, arable fields, bryophytes, Central European endemic, distribution modelling, diversity, expansion, key to determination, *Notothylasorbicularis*, SEM micrographs

## Abstract

In 2023, field research in south-western Poland led to the noteworthy discovery of two hornworts: *Notothylasorbicularis*, a species previously unrecorded in this country, and the rediscovery of *Anthocerosneesii* for the Polish bryoflora. These findings are significant as they suggest a response to climate change, which is facilitating the range expansion of hornworts within Central Europe. Detailed descriptions of the new localities for both species are provided, highlighting the specific environmental conditions and habitats where they were found. Distribution maps for *Notothylasorbicularis* and *Anthocerosneesii* in Poland are provided, as well as SEM micrographs of spores. Additionally, a key to the identification of Polish hornwort species is also included. Furthermore, a model projecting the potential future spread of these hornworts within Poland and the broader Central European region is presented. This model considers climatic variables and habitat availability, offering insights into possible range shifts. This study contributes to the growing body of evidence that climate change is a driving factor in the redistribution of bryophytes.

## ﻿Introduction

Hornworts (Anthocerotophyta), as the sister group to liverworts and mosses, are critical in understanding the evolution of key land plant traits (Frangedakis et al. 2021, 2023). Approximately 250 species of hornworts exist worldwide (Villarreal et al. 2010, 2012; Garcia et al. 2012; Peng and Zhu 2013). They are characterized by their dorsiventral thalli and horn-like sporophytes. In temperate climates, hornworts grow terrestrially, preferring open, moist, or shaded places with nutrient-rich soils (Glime 2017). Especially in the northern hemisphere, they are frequently found in agricultural landscapes, such as stubbled fields, where regular soil disturbance provides the exposed substrate they require (Bisang et al. 2021a, 2021b). These conditions are prevalent in areas subjected to ploughing and other forms of soil turnover, creating the bare ground necessary for hornwort colonization (Bisang 1998; Bisang, Bergamini 2020; Schuster 1992; Villarreal et al. 2010). These plants are often among the first colonizers in these environments, taking advantage of the lack of competition and the availability of light and moisture. In addition to agricultural settings, hornworts commonly inhabit other types of open soil environments, including paths, ditches, and riverbanks, where periodic disturbance or water flow maintains the open soil conditions (Glime 2017). Ecologically, hornworts contribute to soil stabilization and the early stages of soil formation (Bisang 1998). Their presence can influence soil microbial communities and nutrient cycling, as their thalli provide a substrate for microorganisms, and their decomposition contributes organic matter to the soil. The symbiotic relationship between hornworts and cyanobacteria, which fix atmospheric nitrogen, also enhances soil fertility, benefiting other plant species that follow in succession (Bisang 1998; Bisang et al. 2009).

Hornworts are often overlooked because of their small size, the seemingly uninteresting habitat in which they grow, and their short life cycle, which means that they are often only seen for a short period during the year (Bisang 2004). As a result, distributional data for these plants are often absent from relatively large areas. However, to better understand their spread and ecology, we can utilize the results of various floristic reports and ecological surveys. These studies highlight the niche preferences of hornworts and the factors influencing their distribution.

## ﻿History of findings of hornworts in Poland

The history of the study of hornworts in Poland is quite confusing, reflecting the complicated and chequered taxonomic and nomenclatural history of European species of this group of bryophytes. In older literature (Limpricht 1876; Błoński 1888; Rejment-Grochowska 1950) there are only two species reported from Poland classified in the one genus of *Anthoceros* L., namely *A.punctatus* L. and *A.laevis* L. Szweykowski (1958), in his fundamental work on the liverworts in Poland, based on the taxonomy of these bryophytes as taken from the opus of Müller (1906−1911, 1912−1916), reported three species of *Anthoceros* from Poland, *A.crispulus* (Mont.) Douin, *A.laevis* and *A.punctatus*. In turn, Rejment-Grochowska (1966), in the first volume of the Flora of Polish liverworts, based her treatment of the Anthocerotophyta on the study by Proskauer (1958) and provided four species from the country classified into two genera, namely *Phaeoceros* Prosk. (*Ph.laevis* (L.) Prosk.) and *Anthoceros* (*A.punctatus*, *A.crispulus* and *A.neesii* Prosk.). In addition, she provided a description and illustration of *Notothylasorbicularis* (Schwein.) Sull., a species and genus found in the neighbouring countries of Germany and the Czech Republic (then Czechoslovakia) close to the border with Poland, suggesting it would very likely be found in Poland. Finally, Koła and Turzańska (1995) reported five species of hornworts from Poland, namely *Anthocerospunctatus*, *A.neesii* and *A.agrestis* Paton (= *A.punctatus* auct.) and *Phaeoceroslaevis* and *Ph.carolinianus* (Michx.) Prosk.

In the most recent critical lists of hornworts in Poland, Szweykowski (2006), Klama (2006a, 2006b) and Klama and Górski (2018) recognised only three species of these bryophytes from the country, belonging to the genera *Anthoceros* (*A.agrestis* and *A.neesii*) and *Phaeoceros* (*Ph.carolinianus*). According to Szweykowski (2006), the occurrence of *Ph.laevis* in Poland is very doubtful, and a revision of all available herbarium materials so named showed that they actually belonged to *Ph.carolinianus*. Similarly, specimens published and deposited in herbaria under the name *Anthocerospunctatus* actually represented *A.agrestis*. This species was common throughout the country half a century ago, but now is very rare or locally absent in the central and northern lowlands and is more frequent only in the foothills of the Carpathians and the Sudetes in the south of the country.

The knowledge about the occurrence of *Anthocerosneesii* in Poland is relatively poor. It was first recognized as a separate taxon, Anthocerospunctatusf.monocarpus Nees, by Nees von Esenbeck (1838) who described it on the basis of the material he had collected from Grodna hill in the village of Staniszów [*German* Stohnsdorf am Stangenberg] in the Jelenia Góra Basin at the foothills of the Giant Mountains (*Polish* Karkonosze, *Czech* Krkonoše, *German* Riesengebirge) in Lower Silesia in SW Poland. This taxon was actually first collected in the Czech Republic by Corda (1829), who illustrated it in detail as *A.punctatus*. However, Nees von Esenbeck (1838) demonstrated that all illustrations by Corda (1829) but one showing the habit of this hornwort actually represented the form of this species described by himself from specimens collected within the present borders of Poland.

Proskauer (1958) examined the original material of Anthocerospunctatusf.monocarpus in the Nees von Esenbeck herbarium which is apparently housed in STR (Grolle 1976; Stafleu and Cowan 1981) and concluded that this form indeed deserved the status of a separate species. As a result, he raised the form described by Nees von Esenbeck (1838) to the rank of species, which he named *Anthocerosneesii* and designated the original specimen of this form examined by himself as the type, i.e. the holotype. Unfortunately, this is not a completely correct interpretation, as Nees von Esenbeck (1838) also cited Corda’s (1829) illustration in the protologue, so lectotypification is necessary in this situation. According to Art. 9.10 of the current ICN (Turland et al. 2018), Proskauer’s citation of Nees von Esenbeck’s specimen as the holotype is an error to be corrected to lectotype.

Apart from the type specimen, Proskauer (1958) cited three additional speci­mens of *Anthocerosneesii* collected in Poland, unfortunately, without any details regarding collector(s), dates, and herbaria in which they were located. Two specimens were collected in the Jelenia Góra Basin in Lower Silesia in close proximity of the type locality, namely in Malinnik near Cieplice Śląskie Zdrój [*German* Herischdorf bei Warmbrunn] and by the road to the Krzyżna Góra village [*German* am Wege nach Kreuzberg], and the other specimen was collected in the village of Rusinowo [*German* Ruschendorf] in the Wałcz Lakeland in West Pomerania in NW Poland.

After its inception, *Anthocerosneesii* was recorded only once at the type locality from the farmland around the Staniszów village and not far from this place, in the hamlet of Wilcza Poręba in Karpacz in the Karkonosze (Koła and Turzańska 1993). At all sites *A.neesii* co-occurred with other ephemeral bryophyte species typical of habitats of disturbed arable fields. However, the species has not been rediscovered in Poland in the past three decades and therefore it was initially classified as rare (R) (Klama 2006b), but in the latest Red List of Polish liverworts and hornworts *A.neesii* is considered to be critically endangered (CR category) in the country (Klama and Górski 2018). Interestingly, in the European checklist of bryophytes, Hodgetts and Lockhart (2020) ignored the latter Red List and continued to treat *A.neesii* as extremely rare (R) in Poland, but not critically endangered. It is worth noting that despite intensive searches, no historical specimens of this species were found in the main Polish bryological herbaria.

The present study provides data on the rediscovery of *Anthocerosneesii* at two new localities in Lower Silesia in SW Poland. Additionally, the information about the first discovery of *Notothylasorbicularis* in Poland is provided, thus confirming the anticipation of its occurrence in the country expressed by Rejment-Grochowska (1966).

## ﻿Material and methods

### ﻿Plant material and description of the localities

On October 7 and 8, 2023, a bryophyte collection expedition was conducted on several arable and stubble fields in the southern part of the Lower Silesian Voivodeship in Poland. The primary motivation was to gather fresh material of bryophytes for a bryological course for students at Opole University. However, in addition to finding common species of ephemeral bryophytes, two very interesting species of hornworts were discovered. After a preliminary examination in the field, the material was collected and studied in detail in the laboratory. In addition to using classic optical microscopes (Olympus SZ61 and Olympus BX53F), SEM microscopy (Jeol SEM microscope) was also employed to study the surface and ornamentation of the spores in detail, which are crucial for distinguishing hornworts, especially within the genus *Anthoceros*. The distribution of the hornwort species in Poland was plotted on maps in the ATMOS grid square system (Ochyra and Szmajda 1981). The specimens are stored in the OSTR and KRAM herbaria.

**Figure 1.**
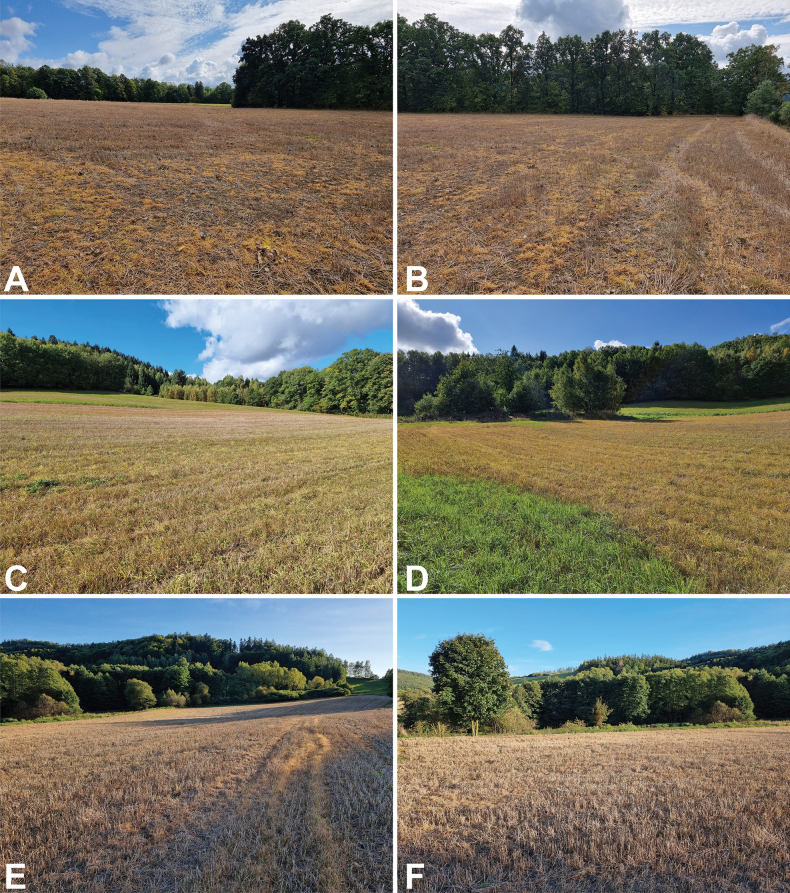
A view of the localities where the hornworts were recently collected **A, B** loc. 1 (between the villages of Szalejów Dolny and Szalejów Górny) **C, D** loc. 2 (near the village of Wambierzyce), and **E, F** loc. 3 (near the village of Mielnik). Photographs were taken by Vítězslav Plášek (7–8 Oct 2023).

A list of localities where the hornworts were recently collected (Fig. 1A–F):

1. S Poland, Lower Silesian Voivodeship, Central Sudetes, Kłodzko Basin, 5 km W of Kłodzko town, between Szalejów Dolny and Szalejów Górny villages, stubble field near the national road No. 8, on open soil near a small forest, 359 m a.s.l., 50°25'53.0"N, 16°34'13.8"E, ATMOS grid square Fb−25, 7 Oct 2023, leg. V. Plášek (Fig. 1A, B).
2. S Poland, Lower Silesian Voivodeship, Central Sudetes, Kłodzko Basin, 12 km WNW of Kłodzko town, 3 km SE of Wambierzyce village, small stubble field near the provincial road No. 388, on open soil, 450 m a.s.l., 50°28'06.7"N, 16°28'33.8"E, ATMOS grid square Fb−15, 7 Oct 2023, leg. V. Plášek (Fig. 1C, D).
3. S Poland, Lower Silesian Voivodeship, Western Sudetes, Karkonosze (Giant Mountains), 8 km S of Kłodzko town, 250 m NE of Przełęcz Mielnicka (=Mielnik pass) in the small village of Mielnik, small stubble field near the national road No. 33, on open soil near a small forest, 382 m a.s.l., 50°21'14.6"N, 16°40'08.2"E, 8 Oct 2023, leg. V. Plášek (Fig. 1E, F).

### ﻿Modelling

Maxent version 3.4.4 software (Phillips et al. 2024) was used to calculate the individual models. The R (R Core Team 2024) and QGIS (QGIS Development Team 2024) programs were used for data preparation and analysis.

### ﻿Species occurrence data

For our analysis, we utilized a comprehensive set of incidence data sources. We incorporated field observation data from the Czech Republic, Germany, and Austria, which were compiled through the review of herbarium specimens, literature excerpts, and using data from national bryophyte distribution databases. Additionally, we integrated data for *Anthocerosneesii* and *Notothylasorbicularis* sourced from the Global Biodiversity Information Facility (GBIF) [https://www.gbif.org/]. These data were accessed via the “sp_occurrence” function within the “geodata” package (Hijmans et al. 2023). Specifically, the data included occurrences for *Anthocerosneesii* (GBIF.org, accessed January 15, 2024, GBIF Occurrence Download: https://doi.org/10.15468/dl.hdc36q) and *Notothylasorbicularis* (GBIF.org, accessed January 15, 2024, GBIF Occurrence Download: https://doi.org/10.15468/dl.zg9vfv).

To address potential sampling biases and errors in the GBIF data, we employed multiple cleaning approaches from “CoordinateCleaner” (Zizka et al. 2019). Additionally, we mitigated sampling bias using the “spThin” package (Aiello-Lammens et al. 2015) in R (R Core Team 2024). With a thinning parameter (“thin.par”) set to 2 km (after testing) and restricted to the geographical extent of (5.8667, 24.1333, 46.3167, 55.05), we obtained 50 samples of *Anthocerosneesii* and 62 samples of *Notothylasorbicularis* for subsequent analysis (Table 1).

**Table 1.** Numbers of records for different datasets.

Species	Time Period	GBIF data After Cleaning	Bryol. Coll.	Overall	Data After Thinning (2 km)
***Anthocerosneesii***	1980–2010	1	42	43	20
	2011 =>	1	48	49	30
***Notothylasorbicularis***	1980–2010	1	88	89	36
	2011 =>	7	37	44	26

### ﻿Environmental layers and variable selection

Nineteen environmental variables (bio1–bio19) at a resolution of 30 seconds (~1 km^2^) were downloaded from the CHELSA dataset (Karger et al. 2017), covering the historical period from 1980 to 2010. For the current/future period (2011–2040), the same 19 environmental variables were obtained from the CHELSA dataset using CMIP6 Global Circulation Models (GCMs), namely GDFL-ESM4 and IPSL-CM6A-LR, under two Shared Socio-economic Pathways (SSPs) 126 and 585.

This publication also utilized information from the European Union’s Copernicus Land Monitoring Service, specifically the Land Cover data for the years 2000 (LC2000) and 2018 (LC2018). These datasets were selected to correspond with the two distinct time periods (LC2000 for 1980–2010 and LC2018 for 2011–2040). The Land Cover data provide detailed information categorized into 15 distinct classes based on updated Land Cover illustrated nomenclature guidelines (Kosztra et al. 2017), ensuring compatibility with Maxent modelling by limiting the number of categories.

To maintain consistency with CHELSA layers, the Land Cover data were resampled to match their dimensions using the “resample” function from the “raster” library (Hijmans 2024) with the nearest neighbor method. From the initial set of 19 environmental variables and two land cover (LC) variables, we eliminated those showing high collinearity using the “vifstep” function from the “USDM” package (Naimi et al. 2014) with a threshold of 0.9. This process yielded a set of 8 uncorrelated variables for each model (bio1, bio3, bio4, bio8, bio9, bio14, bio15, LC2000, or LC2018) (see Table 2).

**Table 2.** Uncorrelated environmental variables used in Maxent modelling.

SHORTNAME	LONGNAME	EXPLANATION
**bio1**	Mean annual air temperature	Mean annual daily mean air temperatures averaged over 1 year.
**bio3**	Isothermality	Ratio of diurnal variation to annual variation in temperatures.
**bio4**	Temperature seasonality	Standard deviation of the monthly mean temperatures.
**bio8**	Mean daily mean air temperatures of the wettest quarter	The wettest quarter of the year is determined (to the nearest month).
**bio9**	Mean daily mean air temperatures of the driest quarter	The driest quarter of the year is determined (to the nearest month).
**bio14**	Precipitation amount of the driest month	The precipitation of the driest month.
**bio15**	Precipitation seasonality	The coefficient of variation is the standard deviation of the monthly precipitation estimates expressed as a percentage of the mean of those estimates (i.e. The annual mean).
**LC2000**	CORINE land cover 2000	The pan- European CORINE land cover inventory for 44 thematic classes for the 2000 reference year.
**LC2018**	CORINE land cover 2018	The pan-European CORINE land cover inventory for 44 thematic classes for the 2018 reference year.

**Note**: Throughout all different time periods, scenarios, and SSPs, the same variables were identified as uncorrelated. The only variation lies in the Land Cover (LC) data utilized for the different time periods, either LC2000 or LC2018, in conjunction with environmental data.

LC2000 and LC2018 data were assessed in combination with bio1-19 sepa­rately, considering potential temporal differences. For each variable combination (across different time periods, scenarios, and SSPs), a distinct Maxent model was constructed. This strategy ensures tailored models for specific conditions, capturing nuances and enhancing the accuracy and interpretability of results compared to utilizing a single model and reprojecting it onto various conditions.

### ﻿Background points and background area

The study area encompasses Central Europe, defined by the geographical coordinates (5.8667, 24.1333, 46.3167, 55.05), predominantly covering the territories of the Czech Republic, Austria, Germany, and Poland. This region was selected for its ecological significance and alignment with our research objectives.

To maintain spatial consistency throughout the modelling process, we ge­nerated 10,000 random background points confined to the entire study area, following the methodology outlined by Phillips and Dudík (2008). This approach of selecting background points from the entire study area mitigates potential issues associated with reprojection to a different territory than that used for model creation.

### ﻿Used algorithm

Maxent, a machine-learning software program, was utilized for species distribution modelling. It calculates raw probability values for each pixel within the study region and is widely acknowledged for its efficacy in predicting species distributions (Elith et al. 2006).

### ﻿Model settings and evaluation

Maxent was employed with varying model complexities to strike a balance between model fit and overfitting. Subsequently, the jackknife method, employing jackknife cross-validation with n-1 folds, was utilized for model complexity estimation. This approach proves advantageous for species with limited occurrence data, facilitating a robust evaluation of model performance across different complexities (Pearson et al. 2007; Shcheglovitova and Anderson 2013).

Model assessment relied on the Area Under the Curve (AUC) metric, offering a comprehensive evaluation of the model’s discriminative ability between suitable and unsuitable habitats across various threshold values. Following the tuning process and subsequent testing, default settings for Feature Class and Regularization were retained, as they exerted minimal impact on the model.

## ﻿Results

### ﻿New finds of *Notothylasorbicularis* and *Anthocerosneesii* in Poland

During the field visit on 7−8 October 2023, two notable findings of hornworts were made in Poland. Firstly, *Notothylasorbicularis* was discovered for the first time in Poland. Secondly, the occurrence of *Anthocerosneesii* in this country was reconfirmed after 36 years. *Notothylasorbicularis* was collected at localities No. 1 and No. 2, while *A.neesii* was found at localities No. 2 and No. 3 (for locality details, see the Material and methods chapter). Populations of both species were relatively abundant, each consisting of several dozen fertile plants. Along with these interesting species, other bryophytes were recorded (listed in alphabetical order): *Anthocerosagrestis*, *Amblystegiumserpens* (Hedw.) Schimp., *Barbulaunguiculata* Hedw., *Brachytheciumrutabulum* (Hedw.) Schimp., *Bryumargenteum* Hedw., *B.klinggraeffii* Schimp. *ex* Klinggr., *B.rubens* Mitt., *Dicranellaschreberiana* (Hedw.) Dixon, *D.staphylina* H.Whitehouse, *Fissidenstaxifolius* Hedw., *Ricciasorocarpa* Bisch., *Tortulaacaulon* (With.) R.H.Zander and *T.truncata* (Hedw.) Mitt.

To prevent potential confusion regarding hornwort species, we provide a brief summary of the most important diagnostic features of *Notothylasorbicularis* and *Anthocerosneesii* with a special reference to the shape and sculpture of the spores (Fig. 3) and a key to the determination of all four species of hornworts occurring in Poland. Moreover, all literature and herbarium records of these two species are presented and their distribution is mapped (Figs 4, 5).

#### ﻿*Notothylasorbicularis*

(Fig. 2A)

The species is characterized by small, rosette-like, flat, prostrate, and round thallus, typically measuring 5–7 (up to 12) mm in diameter. They are dark green, and irregularly lobed at the edge, 6–10 cells thick medially and thinning 2–3-stratose toward margins. Small colonies of cyanobacteria of the genus *Nostoc* Vaucher *ex* Bornet & Flahault are visible in the thallus. The species is monoicous, typically with 2−6 club-shaped antheridia in each cavity. The involucres are scattered near thallus margins, often paired, and they bear capsules that are decumbent on the thallus and project over the edge. The capsules are approximately 1 mm long and oblong-ovate in shape, with a reduced or absent columella. The pseudoelaters are unicellular. The spores are golden-yellow to yellow-green, measuring 35–45 µm in diameter, delicately vermiculate with both proximal and distal faces appearing virtually smooth (Fig. 3J−L). In the size and sculpture the spores of *N.orbicularis* somewhat resemble the spores of *Phaeoceroscarolinianus* in which the proximal faces usually appear nearly smooth but the distal faces are finely echinulate to verruculose (Fig. 3G−I).

**Figure 2.**
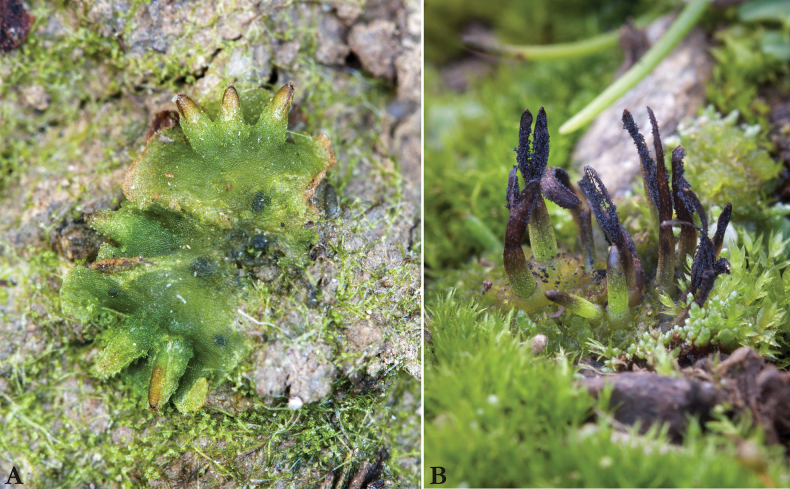
Detail view of the hornworts **A***Notothylasorbicularis***B***Anthocerosneesii*. Photographs were taken by Štěpán Koval.

**Figure 3.**
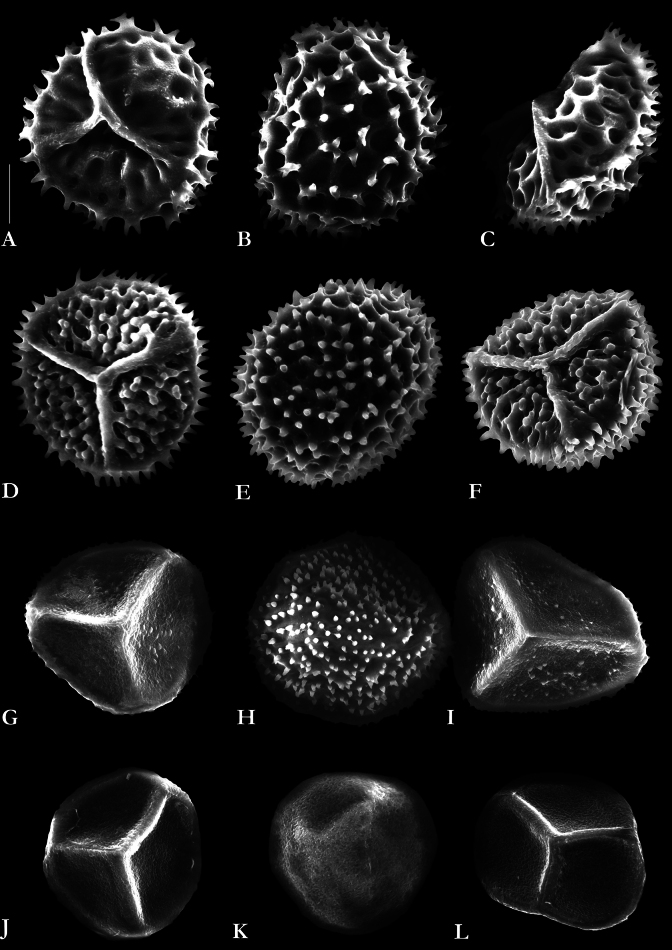
SEM photographs of the spores of hornworts occurring in Poland **A–C***Anthocerosagrestis***D–F***Anthocerosneesii***G–I***Phaeoceroscarolinianus***J–L***Notothylasorbicularis*. Photographs were taken by Vítězslav Plášek. Scale bar: 10 μm.

**Figure 4.**
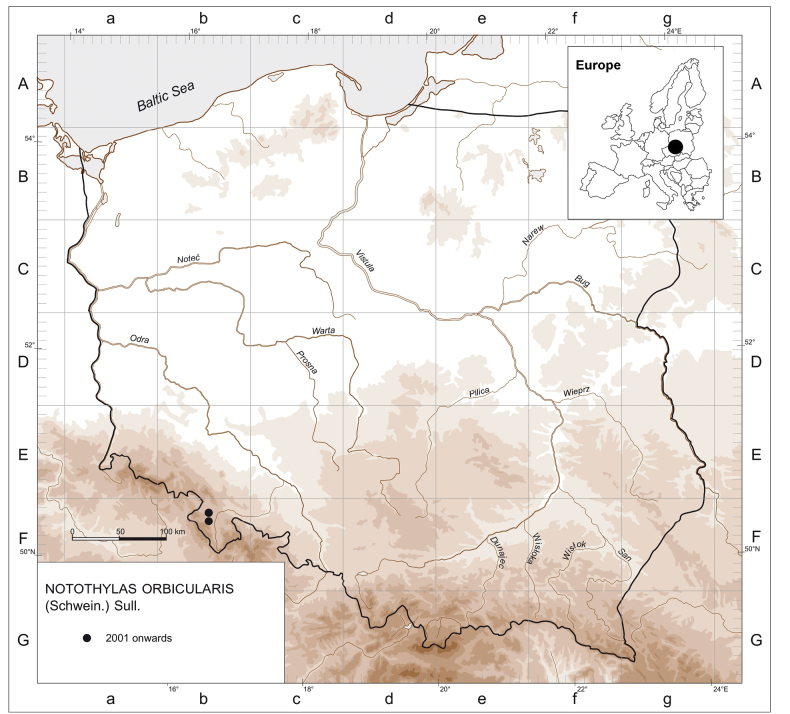
Distribution map for *Notothylasorbicularis* in Poland. Inset: The location of Lower Silesia in Europe.

*Notothylasorbicularis* is a rare species of hornwort in Central Europe, occurring in Germany, Austria, the Czech Republic and now recorded in Poland. According to T. Pócs and A. Sass-Gyarmati, Eger, and P. Širka, Zvolen, this species is, respectively, absent in Hungary and Slovakia. In Poland, it is currently known only from two following localities in the Kłodzko Basin in the Central Sudetes (Fig. 4), although it is very likely that careful field studies will yield additional records of this species in this region. In Europe, the species is otherwise reported from Italy, and Croatia (Rimac et al. 2019; Hodgetts and Lockhart 2020). It is placed in the IUCN European Red List of Mosses, Liverworts and Hornworts in the category of Endangered B2ab (ii, iii, v) (Hodgetts et al. 2019) and is listed on Annex II (animal and plant species of Community interest whose conservation requires the designation of special areas of conservation) of EU Habitats Directive (European Community 1992).

### ﻿List of localities of *Notothylasorbicularis* in Poland

**Fb−15** Central Sudetes, Kłodzko Basin: 3 km SE of the Wambierzyce village, small stubble field near the provincial road No. 388, on open soil, alt. 450 m a.s.l., 7 Oct 2023, leg. V. Plášek (OSTR #8301, KRAM B-278059).

**Fb−25** Central Sudetes, Kłodzko Basin: between the villages of Szalejów Dolny and Szalejów Górny, on open soil on a stubble field near the national road No. 8, alt 359 m a.s.l., 7 Oct 2023, leg. V. Plášek (OSTR #8302, KRAM B-278060).

#### ﻿*Anthocerosneesii*

(Fig. 2B)

Unlike the more frequently occurring species *Anthocerosagrestis*, *A.neesii* is a very small plant, with rosette-like thalli up to 5 mm in diameter. They are 3–4 cells thick medially and arched on the upper surface. The species is monoicous, typically with numerous club-shaped antheridia, up to 60 μm long, in each cavity. The capsules are somewhat club-shaped, brown distally at maturity, mostly 3−7 mm long and 0.3−0.4 mm wide, with a distinct columella. The pseudoelaters are formed of two to several short cells. The mature spores are blackish-brown, 45−54 μm in diameter, with spinulate-blunt protuberances on the proximal faces and simple spines on the distal faces (Fig. 3D−F). The sculpturing of the spores immediately differentiates *A.neesii* from *A.agrestis* which has an almost smooth proximal face with indentations and a distal face covered with forked spines (Fig. 3A−C).

**Figure 5.**
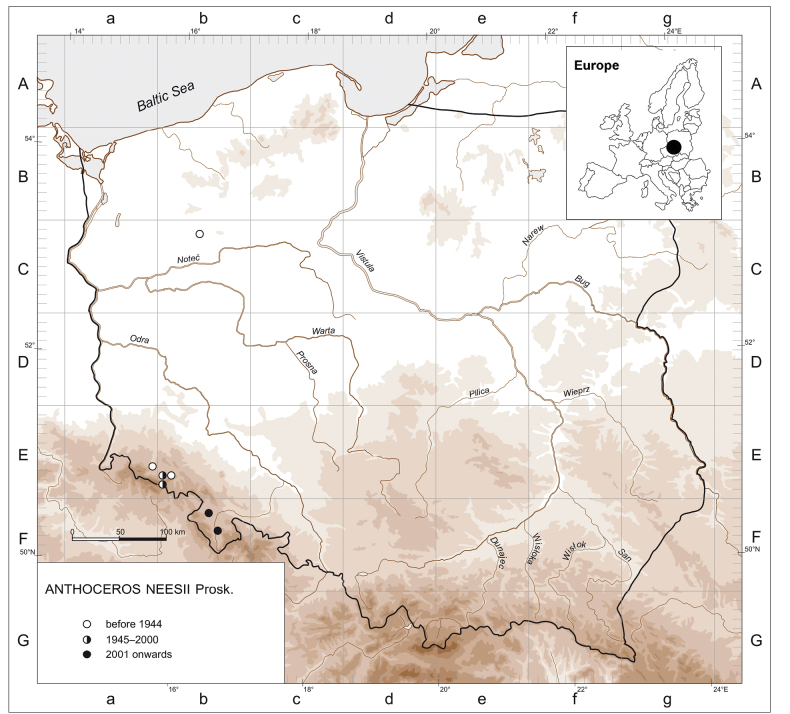
Distribution map for *Anthocerosneesii* in Poland. Inset: The location of Lower Silesia in Europe.

*Anthocerosneesii* is a rare and widely scattered Central European endemic species which is placed in the IUCN European Red List of Mosses, Liverworts and Hornworts in the category of Endangered B2ab(iii) (Bisang et al. 2019; Hodgetts et al. 2019). Apart from Poland, it is known from the Czech Republic, Germany and Austria only (Hodgetts and Lockhart 2020). In Poland, the species has been recorded in seven grid squares in the ATMOS system for mapping bryophytes (Ochyra and Szmajda 1981). In three grid squares it was recorded prior to 1944, in two in the second half of the twentieth century, and in the other two in 2023 (Fig. 5). All localities but one are situated in the Sudetes in the south-western part of the country and only one historical discovery originates from the northern lowlands in West Pomerania. It is worth noting that *A.neesii* was rediscovered in the early 1990s at the type locality in the Jelenia Góra Basin in Lower Silesia and, additionally found at one locality in the Giant Mountains (Koła and Turzańska 1993). However, these discoveries were ignored by the authors of the treatment of *A.neesii* in the IUCN Red List of Threatened Species 2019 (Bisang et al. 2019), who indicated the occurrence of this species in Poland to be “uncertain”.

### ﻿List of localities of *Anthocerosneesii* in Poland

**Cb−14** South Baltic Lakelands, South Pomeranian Lake District, Wałcz Lakeland: Rusinowo (Proskauer 1958).

**Ea−69** Western Sudetes, Jelenia Góra Basin: District Malinnik of Jelenia Góra east of Cieplice Śląskie Zdrój District (Proskauer 1958).

**Eb−70** Western Sudetes, Jelenia Góra Basin: Grodna Hill in Staniszów (Proskauer 1958); Staniszów (Koła and Turzańska 1993)

**Eb−71** Western Sudetes, Krzyżna Góra in the Góry Sokole [= Falcon Mountains] massif east of Jelenia Góra (Proskauer 1958).

**Eb−80** Western Sudetes, Karkonosze: Wilcza Poręba in Karpacz (Koła and Turzańska 1993).

**Fb−15** Central Sudetes, Kłodzko Basin: 3 km SE of the Wambierzyce village, small stable field near the provincial road No. 388, on open soil, 450 m a.s.l., 7 Oct 2023, leg. V. Plášek (OSTR #8303, KRAM B-278061).

**Fb−25** Central Sudetes, Kłodzko Basin: between the villages of Szalejów Dolny and Szalejów Górny 5 km west of Kłodzko town, stable field near the national road No. 8, on open soil near a small forest, 359 m a.s.l., 7 Oct 2023, leg. V. Plášek (OSTR #8304, KRAM B-278062).

### ﻿Key to identification of the hornworts species in Poland

**Table:** 

1	Mature spores blackish-brown, distal faces frequently spinose-dentate (*Anthoceros*)	**2**
–	Mature spores golden-yellow, yellow-green to yellow-brown, distal faces smooth or granular-tuberculate (*Notothylas* or *Phaeoceros*)	**3**
2	Capsules 10−30 mm long, proximal spore faces almost smooth, with indentations, distal faces forming forked spines^*^	***Anthocerosagrestis***
–	Capsules 3−7 mm long, proximal spore faces with spinulate-blunt protuberances, distal faces forming simple (not forked) spines	***Anthocerosneesii*** (Fig. 2B)
3	Capsules decumbent, approximately 1–3 mm long, oblong-ovate; co­lumella reduced or absent; pseudoelaters unicellular; both proximal and distal spore faces smooth	***Notothylasorbicularis*** (Fig. 2A)
–	Capsules erect, more than 1 cm long, with a bristle-like columella; pseudoelaters multicellular, curved; proximal spore faces almost smooth, distal faces verruculose or echinate	***Phaeoceroscarolinianus***

### ﻿Maxent modelling results

**Figure 6.**
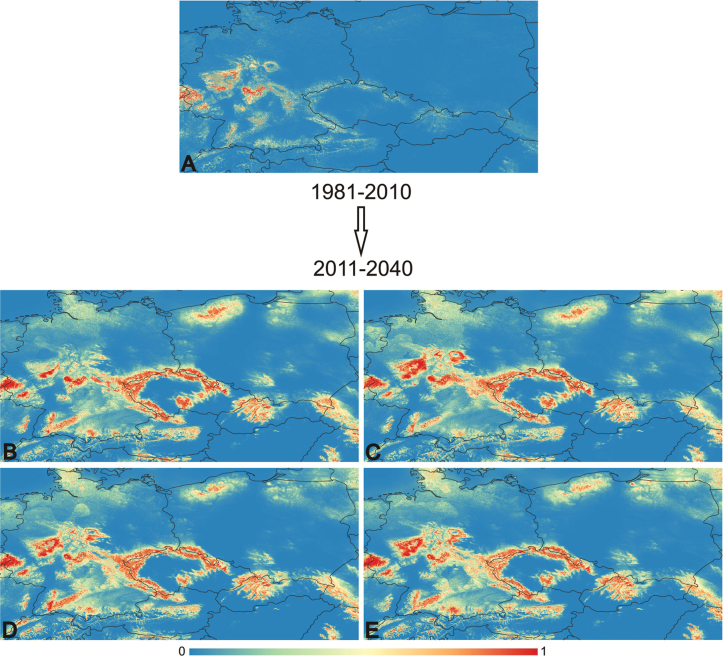
This figure displays the probabilities generated by Maxent across different time periods, GCMs, and SSPs for the species *Anthocerosneesii*. The colour gradient ranges from blue (representing low probabilities, close to 0) to red (representing high probabilities, close to 1). The state borders are outlined for reference. For explanation of the maps **A–E** and detailed values see Table 3.

**Figure 7.**
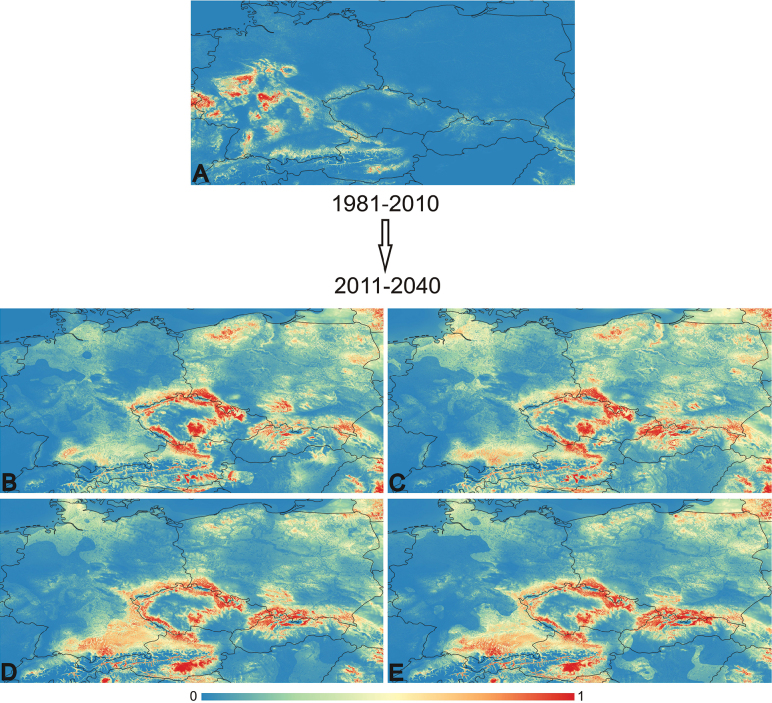
This figure displays the probabilities generated by Maxent across different time periods, GCMs, and SSPs for the species *Notothylasorbicularis*. The colour gradient ranges from blue (representing low probabilities, close to 0) to red (representing high probabilities, close to 1). The state borders are outlined for reference. For explanation of the maps **A–E** and detailed values see Table 3.

In this section, we outline the outcomes of various Maxent models conducted for each studied species across different scenarios (Figs 6, 7). Additionally, we provide average AUC results and highlight the most significant variables identified for each scenario.

**Table 3.** Average AUC values for each species and model.

Model	Average AUC	
	*Anthocerosneesii*	*Notothylasorbicularis*
1980–2010	0,960 (Fig. 6A)	0,955 (Fig. 7A)
2011–2040_gfdl-esm4_ssp126	0,929 (Fig. 6B)	0,863 (Fig. 7B)
2011–2040_gfdl-esm4_ssp585	0,933 (Fig. 6C)	0,829 (Fig. 7C)
2011–2040_ipsl-cm6a-lr_ssp126	0,938 (Fig. 6D)	0,853 (Fig. 7D)
2011–2040_ipsl-cm6a-lr_ssp585	0,934 (Fig. 6E)	0,855 (Fig. 7E)

The following values present estimates of the relative contributions of the variables to the final Maxent model (in %) for different time period, GCMs and SSPs -only variables with contributions to the final model >5% were left in the final models (average contributions in Table 4):

**Table 4.** Average relative contribution of the variables calculated from all models for each species to the final models.

Variable	*Anthocerosneesii*	*Notothylasorbicularis*
**bio14**	25.86	31.22
**bio15**	23.24	8.54
**LC(2000 or 2018)**	19.9	17.42
**bio1**	16.38	31.4
**bio8**	14.58	11.62

*For this purpose, LC2000 and LC2018 were calculated together.

*Anthocerosneesii*:

1980–2010: bio8 – 34.5, LC – 33.6, bio14 – 20, bio1 – 6.1, bio15 – 5.9; 2011–2040_gfdl-esm4_ssp126: bio1 – 36.6, bio14 – 32.7, LC – 13.2, bio15 – 12.1, bio8 – 5.4; 2011–2040_gfdl-esm4_ssp585: bio1 – 36.7, bio14 – 32.8, LC – 13.7, bio15 – 8.6, bio8 – 8.2; 2011–2040_ipsl-cm6a-lr_ssp126: bio14 – 38.3, bio1 – 36.3, LC – 12.7, bio15 – 7.9, bio8 – 5.7; 2011–2040_ipsl-cm6a-lr_ssp585: bio1 – 40.3, bio14 – 32.3, LC – 13.9, bio15 – 8.2, bio8 – 5.3.

*Notothylasorbicularis*:

1980–2010: bio8 – 35.1, bio14 – 25.3, LC – 23.9, bio15 – 9.6, bio1 – 6; 2011–2040_gfdl-esm4_ssp126: bio14 – 29, bio15 – 24.6, bio1 – 20.2, LC – 17.4, bio8 – 8.8; 2011–2040_gfdl-esm4_ssp585: bio14 – 30.6, bio15 – 28.3, LC – 21.4, bio1 – 13.7, bio8 – 6; 2011–2040_ipsl-cm6a-lr_ssp126: bio15 – 27.5, bio14 – 22.7, bio1 – 21.2, LC – 18.2, bio8 – 10.4; 2011–2040_ipsl-cm6a-lr_ssp585: bio15 – 26.2, bio14 – 21.7, bio1 – 20.8, LC – 18.6, bio8 – 12.6.

## ﻿Discussion

Although *Notothylasorbicularis* is often designated as a cosmopolitan species (Dierßen 2001), it is actually a highly disjunct panholarctic temperate species, with the main center of its occurrence in eastern North America (Schuster 1992), and scattered localities in Central and South Europe, as well as in China and Japan in East Asia (Yamada and Iwatsuki 2006; Peng and Zhu 2013). Outside the Holarctic, it only occasionally penetrates into the tropics, with records from Colombia and Ecuador (Gradstein 2021) and eastern Brazil (Gradstein and Costa 2003) in South America, as well as Uganda and the Democratic Republic of the Congo in mainland Africa (Stieperaere and Matcham 2007; Wigginton 2018; Gradstein 2023), and Madagascar (Ellis et al. 2018). Additionally, the species was discovered in Western Australia and Queensland in Australia (Cargill 2016).

In Europe, the species has been recorded in three Central European countries (Austria, Germany, Czech Republic) and two Southern European countries (Italy, Croatia) (cf. Jack 1898; Ludwig et al. 1996; Saukel and Köckinger 1999; Weddeling 2002; Manzke 2004, 2005; Fischer et al. 2008; Koval and Zmrhalová 2010; Kučera 2011; Köckinger 2017; Rimac et al. 2019; Pöltl et al. 2020). Herein, its occurrence is also documented for Poland. In its fertile stage, *N.orbicularis* is nearly unmistakable in the field due to its distinctively formed capsules. However, the young thallus of this species can resemble *Anthocerosagrestis*. A critical distinguishing feature is that the capsules of *N.orbicularis* grow almost horizontally on the thallus or are partially embedded within it and never grow perpendicularly or obliquely as seen in species of *Anthoceros* or *Phaeoceros*. The capsules are ellipsoidal and cigar-shaped, enclosed in a longitudinally warty involucre throughout the ripening period. Fully mature capsules may exhibit a dark tip, and mature spores are smooth on both sides (Fig. 3J−L).

*Anthocerosneesii* (Fig. 2B) has a limited global distribution, being recorded only in the Czech Republic, Poland, Germany, and Austria (Proskauer 1958; Düll 1983; Grolle 1983; Düll and Meinunger 1989; Koła and Turzańska 1993; Dierßen 2001; Weddeling 2002; Manzke 2004, 2005; Meinunger and Schröder 2007; Fischer et al. 2008; Teuber and Göding 2009; Koval and Zmrhalová 2010; Kučera 2011; Schlüsslmayr 2011; Schröck et al. 2014; Köckinger 2017; Pöltl et al. 2020). Given its distribution, *A.neesii* is classified as a Central European endemic. In the field, *A.neesii* can be initially distinguished from other hornworts by its generally smaller stature. The capsules are short and grow perpendicularly or obliquely to the thallus surface, displaying a significant constriction at the transition to the upper dark part where mature spores are located. However, reliable differentiation, especially from small forms of *A.agrestis*, can only be achieved when mature spores are present, which exhibit distinct microscopic features (Fig. 3D−F). Additionally, there is a small difference in mean spore size (n = 50) between *A.neesii* and *A.agrestis* (Pöltl et al. 2020). According our observation, the spores of *A.neesii* measure 45–54 µm and are slightly smaller than those of *A.agrestis*, which measure 38–62 µm.

For both studied species, an increase in the areas suitable for their occurrence in the future is projected. For *Anthocerosneesii*, these areas are primarily located in the border regions of the Czech Republic, as well as in southern, northern, and central Poland, and central regions of Germany. Additionally, suitable areas are expected in the states of Oberösterreich and Steiermark in Austria, as well as certain regions of Slovakia. For *Notothylasorbicularis*, the projected suitable areas are mainly in the border and central regions of the Czech Republic, with additional suitable areas expected to emerge in Slovakia, Poland, and Austria. However, there is a projected decrease in suitable areas in Germany when comparing the period from 1980−2010 to future projections. Overall, there appears to be an expansion of suitable areas for the spread of both species.

According to the results of our study, climate change is significantly influencing the distribution and expansion of hornworts (Anthocerotophyta) in Central Europe. Rising temperatures and altered precipitation patterns are causing shifts in their traditional habitats (Bates and Preston 2011; van Zuijlen et al. 2024). Warmer temperatures can extend the growing season, potentially allowing hornworts to colonize areas previously too cold for their establishment. However, these advantages are often counterbalanced by increased risks of drought and habitat desiccation (Gignac 2001; Bisang et al. 2021a, 2021b).

Additionally, climate change can lead to alterations in land use patterns, such as changes in agricultural practices and forest management, further impacting hornwort habitats (Dale 1997; Olesen and Bindi 2002; Oliver and Morecroft 2014; van Zuijlen et al. 2024). For example, an increased frequency of intense agricultural activities may create more disturbed soils (Ewert et al. 2005), which can be beneficial for some hornwort species. Conversely, such practices may also result in habitat loss through land conversion. Additionally, the rapid ploughing of harvested fields, especially cereal fields, poses a risk to both species.

Hornworts depend heavily on moisture for their growth and reproduction. Changes in precipitation patterns, particularly reduced summer rainfall and an increased frequency of droughts, can adversely affect their populations (Spinoni et al. 2015). In Central Europe, the variability in seasonal precipitation has led to fluctuating soil moisture levels, impacting hornworts’ ability to thrive in their typical moist and shaded microhabitats. Despite these challenges, some hornwort species may find new niches in disturbed areas created by climate-induced events such as flooding (Madsen et al. 2014). Floodplains and areas prone to periodic waterlogging can provide suitable environments for hornworts, potentially aiding in their expansion. Furthermore, milder winters and reduced snow cover might also facilitate hornwort survival and propagation in regions previously constrained by harsh winter conditions.

Research indicates that climate change may also affect the symbiotic relationships hornworts have with cyanobacteria, which are crucial for nitrogen fixation. Changes in temperature and moisture levels could disrupt these symbioses, impacting hornworts’ growth and their role in nutrient cycling within ecosystems (Tylianakis et al. 2008).

The northward migration of certain hornwort species has been observed and is attributed to the warming climate. This shift is a response to changing temperature and precipitation regimes, allowing species to move into new territories where conditions have become more favourable (cf. Zanatta et al. 2020; van Zuijlen et al. 2024). However, this migration is not uniform across all species and depends on their specific ecological requirements (Zanatta et al. 2020). The fact that *A.neesii* is endemic to Europe underscores the significant responsibility of the countries where this species occurs to ensure its protection.

## ﻿Conclusions

The impact of climate change on these hornworts is multifaceted. While certain changes may offer new opportunities for colonization, others pose signi­ficant risks by disrupting their delicate ecological niches. Continuous research and monitoring are crucial to comprehending these dynamics and formulating strategies to mitigate adverse effects on these species.

In conclusion, hornworts represent a distinctive and ecologically significant component of Central Europe’s bryophyte flora. Their distribution is influenced by a combination of climatic, edaphic, and anthropogenic factors. Continued research and conservation efforts are imperative to ensure the persistence of these unique plants amidst ongoing environmental changes.

## Footnotes

* For detailed views of the spore faces of each species, see SEM photographs (Fig. 3A–L).

## ﻿Additional information

### Author ORCIDs

Vítězslav Plášek https://orcid.org/0000-0002-4664-2135

Lukáš Číhal https://orcid.org/0009-0009-2740-1326

Frank Müller https://orcid.org/0000-0001-9482-9423

Martina Pöltl https://orcid.org/0000-0002-3001-058X

Mariusz Wierzgoń https://orcid.org/0000-0002-4809-2202

Ryszard Ochyra https://orcid.org/0000-0002-2541-0722

### Data availability

All of the data that support the findings of this study are available in the main text.
